# Facilitating Exercise Habit Formation among Cardiac Rehabilitation Patients: A Randomized Controlled Pilot Trial

**DOI:** 10.3390/ijerph18126440

**Published:** 2021-06-14

**Authors:** Navin Kaushal, Marie Payer, Béatrice Bérubé, Martin Juneau, Louis Bherer

**Affiliations:** 1 Department of Health Sciences, School of Health & Human Sciences, Indiana University, Indianapolis, IN 47405, USA; 2 Department of Medicine, Université de Montréal, Montréal, QC H3T 1J4, Canada; payer.marie@courrier.uqam.ca (M.P.); martin.juneau.1@umontreal.ca (M.J.); louis.bherer@umontreal.ca (L.B.); 3 EPIC Centre of Montréal Heart Institute, Montréal, QC H1T 1C8, Canada; berube.beatrice.2@courrier.uqam.ca; 4 Centre de Recherche de l’Institut Universitaire de Gériatrie de Montréal, Montréal, QC H3W 1W5, Canada

**Keywords:** physical activity, exercise, habit, dual process, cardiac rehabilitation, acute coronary syndrome, heart community, older adults

## Abstract

Background: The importance of promoting exercise adherence among individuals with acute coronary syndrome (ACS) is imperative. However, challenges in maintaining behavior among ACS patients are also well-documented. Emerging findings in the general population have supported the use of habit-formation techniques, which include incorporating routine consistency and cues, to be effective for facilitating exercise behavior. The effectiveness of habit formation approaches, however, has not been tested on participants with ACS. The purpose of this study was to test the effectiveness of facilitating physical activity habits among patients with ACS in a two-arm, parallel design, randomized controlled pilot trial. Methods: Participants (n = 13) were older adult patients (M age = 64.20, SD = 5.35) with ACS who were referred to a cardiac rehabilitation center. The experimental group attended monthly group meetings from months 1–3 and received phone call follow-ups from months 4–6. Conclusions: The experimental group showed an increase in weekly moderate-to-vigorous level physical activity, M = 228.20 mins (SD = 112.45), compared with the control group, M = 151.17 (SD = 112.22), *d* = 0.61. The experimental condition also showed greater use of routine consistency (experimental: M = 4.60 (SD = 0.548); control: M = 3.76 (SD = 1.62)) and cue usage (experimental: M = 3.60 (SD = 0.471); control: M= 2.60 (SD = 0.398)) over the control condition at the six-month mark. The study supports the effectiveness of habit-building techniques among patients with ACS, with effect sizes ranging from a medium to large magnitude. Findings from this pilot study support a full clinical trial with larger sample size.

## 1. Introduction

Patients who have experienced a myocardial infarction have a fourfold risk of experiencing sudden cardiac death within 30 days compared to the general population [1]. A common precursor to myocardial infarction and sudden cardiac death is acute coronary syndrome, which is a blockage of blood flow to the heart, usually caused by clot formation or plaque rupture in the coronary arteries. Among several determinants that have been identified to prevent the recurrence of cardiac events, physical activity has been identified as one of the strongest preventive measures [2,3,4]. While most cardiac rehabilitation centers include an environment for cardiac patients to exercise, retention remains a global challenge, as dropout rates average close to 60% across 37 countries [5]. Efforts to improve participation can be noted from several empirical trials that have been appraised by recent systematic reviews. Specifically, the reviews identified the importance of using a theoretical framework to test behavior change strategies [6] and a need to improve reporting consistency to enhance the generalizability of findings [6,7,8].

Behavior change interventions that are based on a theoretical framework can help us understand the mechanisms of predicting behavior change [9,10]. Accumulated literature work on theories can help us identify mechanisms that are and are not effective for a particular population group. Research in promoting physical activity primarily resorted to applying social cognition theories to understand this behavior. However, there have been some noted limitations of applying these models to predict physical activity, such as the absence of automatic processes [11,12], and hence, there have been recommendations to consider alternative models that do include this process [11,13,14]. A contemporary approach that acknowledges that our behaviors are the result of conscious (intentional) and automatic (habit) processes is dual process theorizing [15]. A habit can be defined as a discrepancy between thought and behavior (such as performing your morning routine while planning your day ahead) [16]. Individuals develop habits by repeatedly performing a behavior in a stable context [13,17,18]. For instance, a randomized controlled trial among novice exercisers found that the implementation of cues (e.g., gym clothes), in conjunction with keeping a consistent exercise time, provided conducive conditions for the behavior to become predictable, thus requiring less cognitive thought and allowing the behavior to become a habit [19]. We propose the adoption of these methods to patients with acute coronary syndrome to be feasible given the similarities between behavioral requirements, which specifically include being able to safely engage in exercise in a gym environment. To the best of our knowledge, theory-based behavioral research among cardiac rehabilitation patients is very limited, and habit formation techniques have not been tested in any cardiac rehabilitation patients.

The second concern noted in behavioral interventions among cardiac rehabilitation patients includes a lack of defined methodological consistency, which creates challenges when comparing results [8]. For instance, limited clarity in reporting clinical trials hinders replicability, and a lack of justification for prescribed exercise goals results in the variability of interventions, making it a challenge for comparing intervention approaches. The proposed study aimed to improve clarity aspects by prescribing participants to engage in at least 150 mins of moderate-to-vigorous physical activity (MVPA)/week, which is the standard recommendation for patients with cardiac disease proposed by the British Association for Cardiovascular Prevention and Rehabilitation [20].

The purpose of this study was to test if principles of habit formation would be a viable approach for facilitating exercise participation among patients with acute coronary syndrome using a randomized controlled pilot trial design. The primary objective tested if participants in the experimental arm demonstrated greater MVPA time/week compared with the control condition at the primary (month 3) and secondary (month 6) time points of the study. Adherence at cardiac rehabilitation centers tends to be stable during the active support phase from the center, but large attrition is usually observed when individuals need to volitionally continue their physical activity [5]. The selected rehabilitation center for the present study offers training support for the first three months. It was hence hypothesized that participants would not be significantly different at the primary measurement point (month 3), but that they would be significantly different at the secondary timepoint (month 6). Intervention fidelity was tested if the experimental group successfully implemented and applied the advised habit-building variables (consistency and cues), compared with the control condition at month six.

## 2. Materials and Methods

### 2.1. Trial Design

The intervention used a two-arm, parallel design randomized controlled trial (RCT). Participants were randomized to the experimental or the control condition at a 1:1 ratio using Microsoft Excel. The primary and secondary endpoints of the study were months 3 and 6, respectively. The study followed CONSORT reporting guidelines [21]. The study was approved by the Montreal Heart Institute (#20172235) and informed consent was obtained from all participants. The trial has been registered at clinicaltrials.gov: NCT04806841 (last update: 19 March 2021).

### 2.2. Participants

**Figure 1:**
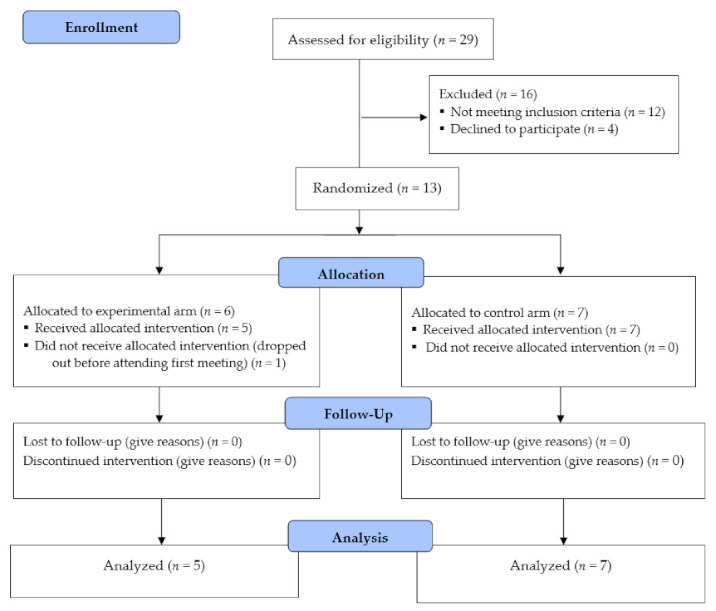
Study Flow diagram.

Table 1 presents participants’ characteristics. Participants (*n* = 13) were patients who were diagnosed with acute coronary syndrome and were referred to the Montreal Heart Institute corresponding rehabilitation center (EPIC Centre). Participants were new members at the rehabilitation center (age 50–84) who were diagnosed with acute coronary syndrome and were not meeting the recommended physical activity guidelines at the time of recruitment [22]. Each new member at the rehabilitation center completed various fitness and cardiovascular measures by a nurse. Upon completion of the measures, the nurse provided the study advertisement to the patients. Individuals who were interested contacted the lead research assistant who discussed the study with the potential participant and presented the consent form. Individuals who agreed to consent were then assessed based on the eligibility criteria, which was a three-step process. Participants were excluded if they experienced a recent valve surgery without a coronary event, non-cardiopulmonary exercise limitation, malignant exercise arrhythmia, and decompensated heart failure. Those who met the eligibility criteria then completed a Montreal Cognitive Assessment (MoCA) [23] and were evaluated based on recommended guidelines [24]. Finally, participants who passed the study eligibility criteria were then required to acquire a notice from their cardiologist to approve their participation in the study. The participant flow chart is depicted in Figure 1.

### 2.3. Intervention

The experimental group attended presentations monthly from months 1–3 and received phone call follow-ups from months 4–6. The first group meeting overviewed the benefits of meeting the recommended physical activity guidelines (150 mins/week of MVPA), followed by guiding the participants on how to establish an (preparatory) exercise habit. Processes in the preparatory phase could include getting ready at home (switching to gym clothes) or gathering workout gear (e.g., water bottle, gym clothes). Participants were then presented with the behavioral and psychological requirements to establish a habit. Previous findings and empirical work have shown that individuals who exercised for a least four days/week for six consecutive weeks established a predictable routine, which used less conscious cognitive resources and thus allowed automatic processes (habits) formation to develop [25]. This was paired with establishing a reminder consistency and cues (psychological components). During the second group meeting, participants shared success strategies and personal experiences on developing their exercise habit. This meeting facilitated group cohesion, as participants were able to relate to one another. The final meeting encouraged the group to discuss and brainstorm ideas on how their habits could be applied to facilitate an overall active lifestyle (such as going for a walk), and they were assisted in generating ideas on where to exercise beyond the gym (indoors and outdoors). Participants were provided with a summary note which highlighted key points in all presentations. Research assistants conducted monthly phone call follow-ups from months 4–6 to address any potential concerns and ask about their experiences. The control arm attended group meetings related to healthy nutrition and diet. Participants in the control arm also had access to the same gym in the rehabilitation center.

### 2.4. Outcomes

Exercise activity time was compared between groups at the primary (three month) and secondary (six month) time points of the study. The kinesiologist on-site monitored participant exercise and assisted participants in completing exercise behavioral time to ensure data validity. The leisure-time exercise questionnaire was used to assess exercise behavior [26]. The scale comprises three open-ended items measuring time (duration and frequency) spent on each type of exercise intensity: mild, moderate, and strenuous. The measure has demonstrated good two-week test–retest reliability for exercise time (*r* = 0.74) and frequency (*r* = 0.80) components [27]. For the purpose of this study, we assessed moderate and vigorous level intensity to reflect the intensity levels from the recommended guidelines [20].

### 2.5. Fidelity Tests: Contextual Predictors

Fidelity tests were incorporated to test if participants in the experimental condition incorporated contextual variables (consistency and cues). Consistency was assessed using an item worded, “How consistently do you exercise at the same time each day? (e.g., exercising every morning at 7 a.m. or exercising daily after lunch).” Cues was assessed using the following item, “I use cues at home to remind me to exercise, such as placing my gym clothes on the bed”. Both items were rated on a five-point Likert scale that ranged from, “1 = strongly disagree” to “5 = strongly agree.” These scales have demonstrated predictive validity in previous work [19]. 

### 2.6. Statistical Methods

Pilot studies are not designed to be sufficiently powered to determine group significance [28,29]. Rather, the purpose of a pilot study is to analyze effect sizes to, hence, determine if a fully powered RCT would be a viable investment to test for significant effects. The present study, hence, used Cohen’s D to identify an effect size difference between groups, followed by eta squared, which measures the proportion of total variance in a dependent variable that is associated with group type (control vs. experimental) based on recommended primers for clinical interpretation [30,31]. The primary outcome was investigated by calculating mean differences in MVPA time at the primary (month three) and secondary (month six) endpoints of the study. The primary outcome was further tested by conducting an ANCOVA to calculate the change in exercise time. The baseline assessment for each variable was treated as a covariate in each ANCOVA model, and the follow-up measures were the dependent variables [32]. Any differences in demographic variables were controlled as covariates. Next, a fidelity check was conducted to test if the experimental group applied habit-building constructs (cues and consistency) by the end of the study (month six). This was investigated by calculating the mean differences of the two constructs, followed by the same procedures described for conducting the ANCOVA.

Power analyses were performed to conduct a full RCT with two groups, and six repeated measures revealed that a sample of 76 participants would be needed to detect significant differences at a small effect size (f^2^ = 0.25) [28]. It has been recommended that a pilot study should be at least 10% of the full trial [29], which approximates to at least eight participants. Missing data analyses were used to handle potential missed measures and data [30,31]. Research assistants were responsible for delivering the intervention, and a dedicated exercise kinesiologist helped all patients perform exercises when needed.

## 3. Results

### 3.1. Participant Flow and Baseline Data

Patients diagnosed with acute coronary syndrome (*n* = 29) showed interest in the study by contacting the research assistant (R1). Twelve participants were excluded as per the eligibility criteria, and four declined to participate. Participants (*n* = 13) were randomized into the experimental (*n* = 6) or the control (*n* = 7) group. One participant dropped out post-randomization, leaving 92% of the participants who completed the study, which fell within the range of a strong trial (80–100% retention) [33].

### 3.2. Missing Outcomes

Missing analysis found that 12.18% of the data was missing. Further investigation using Little’s MCAR test revealed that the data were missing completely at random (χ^2^ = 51.96, DF = 67, *p* = 0.91); hence, it was appropriate to proceed with expectation maximization [34].

### 3.3. Baseline Data 

Participants were 64.20 (SD = 5.35) years of age, and the average BMI was 28.31 (SD = 6.85). The groups were homogenous in baseline variables (Table 1) and did not differ in physical activity time at the baseline, (F (1,12) = 0.125, *p* = 0.73 (η^2^ = 0.01)). The groups were found to be homogenous across all demographic variables, with the exception of participant age, which was, hence, controlled when investigating the study objectives.

### 3.4. Participation and Behavior Change

Group differences analyzed at month three were found to be trivial, (F (3, 12) = 0.89, *p* = 0.38 (η^2^ = 0.10)), *d* = 0.10. The experimental group demonstrated an increase in moderate-to-vigorous level physical activity (MVPA), M = 228.20 mins (SD = 112.45), compared with the control condition, M = 151.17 (SD = 112.22), at the six-month mark, (F (3,12) = 2.42, *p* = 0.16 (η^2^ = 0.23)), with a large magnitude difference, *d* = 0.61.

### 3.5. Fidelity Tests-Contextual Variables

Fidelity tests supported the effectiveness of intervention delivery, as the intervention group was found to successfully apply the contextual variables. Measure scores for practicing regular consistency was higher in the experimental, M= 4.60 (SD = 0.548), compared with the control condition, M = 3.76 (SD = 1.62), at the six-month mark, (F (3,12) = 1.69, *p* = 0.23 (η^2^ = 0.16)), *d* = 1.19. Similarly, scores for reporting cue usage were also higher for the intervention group, M = 3.60 (SD = 0.471), compared with the control condition, M = 2.60 (SD = 0.398), at the six-month mark (F (3,12) = 2.96, *p* = 0.12 (η^2^ = 0.25)), *d* = 1.27. Group differences translated to a large effect size change in implementing routine consistency and cues.

### 3.6. Harms

No participants reported any experiences of harm related to the study.

## 4. Discussion

The purpose of this study was to advance findings in understanding behavioral adherence in cardiac rehabilitation by incorporating recommendations from recent reviews. Consistent with the primary hypothesis, group differences in physical activity time were not found at the midpoint of the study (month three), but the difference in exercise time were of medium magnitude at the end of the study (month six) [31] in favor of the experimental group. As hypothesized, the trivial differences at the midpoint of the study could be attributed to the support offered at the cardiac rehabilitation center. However, similar to gym dropouts during the New Year phase, the novelty of the environment, over time, may become a less salient drive for conscious intention. The difference in engagement in physical activity time at the six-month mark, hence, represents sustainment from the experimental group, as automatic (habit) process likely made it easier to maintain behavior.

The experimental group also showed greater usage of implementing routine consistency of their behavior and using environmental cues, thus corroborating intervention fidelity. The practice of manipulating environmental context to prompt behavior is essentially establishing a plan, which supports planning as a useful mechanism for sustaining exercise behavior [35,36]. However, the plan in the present study was a preparatory ritual as it readied one to exercise, and hence, it was analogous to other preparatory routines that initially require conscious deliberation but become automatic over time (such as an individual’s morning routine). The present findings provide some new notes for the application of habit formation for clinical population groups, specifically for individuals diagnosed with acute coronary syndrome.

Although the study demonstrated feasibility for adopting novel behavioral tactics to individuals with acute coronary syndrome, the findings should be interpreted in a respective scope. The study, overall, provides preliminary evidence of applying a habit formation approach for patients with acute coronary syndrome and supports further research to conduct a larger trial to test habit formation among this demographic.

## 5. Conclusions

Exercise adherence among cardiovascular rehabilitation centers is a well-documented and ongoing challenge [37]. Attributions to this challenge include limited studies that used a theory-based approach and justified methodology as outlined by recent reviews. Incorporating recommendations for methodological design, combined with a theory-based behavioral approach, revealed novel preliminary findings for cardiac rehabilitation patients. In summary, this pilot study supports the efficacy of conducting a full randomized controlled trial to facilitate an exercise habit among patients with acute coronary syndrome.

## Figures and Tables

**Table 1:** Demographic characteristics of the sample.

Characteristic	F or χ^2^	*p*	Control Mean (SD)	Experimental Mean (SD)
				
Age	F = 7.32	0.02	60.43 (5.74)	69.60 (5.86)
BMI	F = 0.816	0.39	26.18 (5.43)	29.83 (7.73)
MVPA	F = 0.125	0.73	71.38 (58.83)	56.80 (84.67)
Female	χ^2^ = 0.80	0.67	5	4
Married/common law	χ^2^ = 0.01	0.92	4	3
>$75,000 Income	χ^2^ = 3.08	0.08	2	4
Completed University	χ^2^ = 1.03	0.52	6	3
Currently Employed	χ^2^ = 1.67	0.29	3	4
				
Overall Health	F = 0.06	0.82	3.29 (0.76)	3.40 (0.89)
Geriatric Depression Scale	F = 2.28	0.16	1.59 (1.37)	4.17 (4.62)
MoCa Score	F = 1.96	0.19	25.93 (1.57)	27.17 (1.72)
*Cardiac-Related Health Symptoms*				
Angina	χ^2^ = 1.53	0.22	7	4
Chest pain or discomfort	χ^2^ = 1.53	0.22	7	4
Upper body discomfort	χ^2^ = 0.069	0.80	6	4
Shortness of breath	χ^2^ = 0.343	0.56	3	3
Palpitations	χ^2^ = 0.069	0.80	6	4
				
Other Health Conditions	F = 0.488	0.50	9.40 (1.67)	7.60 (1.98)

## Data Availability

The data presented in this study are available on request from the corresponding author. The data are not publicly available due to privacy or ethical restrictions.
